# Simplified Chinese lexicon project: A lexical decision database with 8105 characters and 4864 pseudocharacters

**DOI:** 10.3758/s13428-025-02701-7

**Published:** 2025-06-23

**Authors:** Yixia Wang, Yanxue Wang, Qi Chen, Emmanuel Keuleers

**Affiliations:** 1 Tilburg University, Warandelaan 2, Tilburg, 5037 AB Netherlands; 2 South China Normal University, Guangzhou, China; 3 Shenzhen University, Shenzhen, China

**Keywords:** Visual word recognition, Lexical decision, Megastudy, Trial level analysis, Virtual experiments, Simplified Chinese characters, Pseudocharacters

## Abstract

This paper presents the Simplified Chinese Lexicon Project (SCLP), which collects lexical decision data for all 8105 characters in the *List of Commonly Used Standard Chinese Characters* and for 4864 pseudocharacters, which were generated using a novel method that leveraged the hierarchical nature of Chinese characters. We compared the collected data to existing megastudies on Chinese characters, and found that the newly collected data performed similarly in terms of reliability. The comprehensive coverage of simplified Chinese characters in the present study added to the existing studies by allowing for a more fine-grained investigation of the effects of a variety of character attributes on visual processing. We illustrated these advantages by performing virtual experiments on visual complexity and on the interplay between neighborhood size and regularity. Our results indicated that characters with higher visual complexity were harder to recognize, in line with previous findings, while regular characters took longer to process when the neighborhood size was small. In addition, we present a new evaluation of the interaction between character frequency and subcomponent frequency, resulting in a three-way interaction among character frequency, radical frequency, and residual component frequency. Extending the investigation of subcomponent frequency to the analysis of pseudocharacters, we found that the interaction of radical frequency and residual component frequency also modulated pseudocharacter rejection. To support researchers in conducting behavioral experiments or statistical modeling, we provide both trial-level data and experiment materials.

## Introduction

In the last 15 years, the megastudy approach has vastly increased the availability of visual word recognition data for various languages with alphabetic scripts, such as English (Balota et al., 2007; Keuleers et al., 2012), Dutch (Brysbaert et al., year; Keuleers et al., 2010), French (Ferrand et al., 2010), Malay (Yap et al., 2010), Spanish (Aguasvivas et al., 2018; Miguel-Abella et al., 2022), Persian (Nemati et al., 2022), and Hebrew (Stein et al., 2023). Visual word recognition megastudies typically collect response data for a large proportion of a language’s words, allowing a multitude of research questions to be answered without the need to design and execute an ad hoc experiment (Brysbaert et al., 2018; Keuleers & Balota, 2015). The amount of stimuli and their variability offer advantages in terms of statistical power and modeling of effects of continuous variables (Cortese, 2019) and improve the generalizability of findings (Yarkoni, 2022). Analysis of megastudy data has greatly refined our understanding of the effects of word form (Ferrand et al., 2010; Miguel-Abella et al., 2022; Stein et al., 2023), semantics (Keuleers et al., 2012), phonology (Stein et al., 2023; Yap et al., 2010), frequency of usage (Keuleers et al., 2012; Miguel-Abella et al., 2022), effective dimensions (Balota et al., 2007), and reader characteristics (Kuperman & Van Dyke, 2013; Baayen et al., 2016).

### Existing megastudies on Chinese word and character recognition

Similar efforts have been made to collect megastudy data for languages with non-alphabetic scripts, in particular for Chi-nese. There are two closely related variants of the morphosyllabic writing system (DeFrancis, 1989; Gorman & Sproat, 2023) to write Chinese: Hong Kong, Taiwan, and Macau use traditional Chinese characters, while mainland China, Singapore, and Malaysia use simplified characters. The simplified characters, which were first introduced in 1956, require fewer strokes to write and are visually less complex (Cheng, 1975; McBride-Chang et al., 2005).

What makes the study of visual word recognition in Chinese distinctly different is its large character set. While reading in alphabetic writing systems involves a relatively limited set of letters (e.g., 26 in English), the Chinese writing system requires readers to recognize several thousand characters. In almost all cases, these single characters are also words by themselves. In addition, the bulk of Chinese words are bimorphemic (Mok, 2009). Megastudies in Chinese, therefore, often focus on one- and two-character words. While the former is the main focus of the current paper, studies using two-character compounds (Tsang & Zou, 2022; Tse et al., 2017, 2023), are not less informative.

**Table 1:** Overview of megastudies on single character recognition, by task (lexical decision, naming, or handwriting), character variant (traditional or simplified), number of characters (*N*Char), number of participants (*N*Participant), and the average number of characters per participant (unknown for Tsang et al. , (2018), because they use up to 12,578 words with different lengths from one to four

Study	Task	Variant	*N*	*N*	*N*Char per
			Char	Participant	participant
Lee et al. (2015)	LD	traditional	3423	180	570 or 573
Chang et al. (2016)	naming	traditional	3314	140	473 or 476
Liu et al. (2007)	naming	simplified	2423	39	2423
Sze et al. (2014)	LD	simplified	2500	35	2500
Tsang et al. (2018)	LD	simplified	1020	504	unknown
Wang et al. (2020)	handwriting	simplified	1600	205	200

These stimuli are divided into 12 lists, but in each list no clear numbers of their distribution are given.)

Table 1 gives an overview of existing megastudies dealing with single-character recognition. Consistent with the literature on visual word processing in alphabetic languages, the strongest effects found in these studies relate to how often characters are encountered throughout development: characters with higher frequency and familiarity and earlier age of acquisition and age of learning are easier to process (Chang et al., 2016; Lee et al., 2015; Liu et al., 2007; Sze et al., 2014; Tsang et al., 2018).

Taken together, however, the results also show an intricate interplay between a character’s composition and its relation to other characters. Lee et al. (2015); Liu et al. (2007), and Sze et al. (2014) have shown that processing time increases with visual complexity (number of strokes) and results by Liu et al. (2007) and Chang et al. (2016) show that characters that consist of a semantic and a phonetic component are easier to name when the phonetic component agrees with the whole character pronunciation. Characters with multiple pronunciations are harder to recognize (Tsang et al., 2018), but the number of other characters a character shares phonetic components with makes it faster to process a character (Chang et al., 2016). Also, if a character shares the same pronunciation with other characters – its homophones – then it becomes easier to process if those characters also have the same phonetic component (Lee et al., 2015, LDT). However, the number of homophones a character has and the frequency of characters with the same pronunciation do not seem to affect processing Liu et al. (2007). With respect to semantics, the number of characters sharing the same semantic radical does not facilitate naming (Chang et al., 2016), whereas the number of meanings does facilitate recognition for low-frequency characters (Sze et al., 2014). Semantic variables such as concreteness and imageability also facilitate reading (Liu et al., 2007).

#### Limitations of existing studies

Chinese characters are considered to be a closed class (Hockett, 1951; Hoosain, 1992). In other words, while new words can be formed by combining existing characters, no new characters are being created for the sake of creating new words. According to the *List of Commonly Used Standard Chinese Characters*, the official list used in Mainland China (Language and Text Information Management Department, 2013), there are 8105 simplified characters. However, some of these characters are not in widespread use, often being limited to formal texts (Hoosain, 1992). The SUBTLEX-CH corpus (Cai & Brysbaert, 2010), which is based on movie and television subtitles, contains 5936 unique characters, of which 4746 are used more than five times. If anything, this indicates that not all characters are frequently used. However, which characters are known and to what extent cannot be determined based on the corpus data alone. For instance, some characters may correspond to a register that is not often represented in the corpus data. Therefore, determining which characters should be included in a megastudy based on their usage, is not easy. Practically, it is also possible to simply collect megastudy data for *all* Chinese characters. Indeed, the full set of 8105 characters would still be much smaller than the number of words collected in several megastudies in alphabetic languages (e.g., ELP, 40,481 words; BLP, 28,730 words; FLP, 38,840 words). Surprisingly, as Table 1 shows, none of the existing megastudies on Chinese character processing have presented the complete set of simplified characters to participants.

Collecting data on this full set of characters maximizes its research potential. For example, each character has a radical, a component used for dictionary lookup (called *bushou*  in Chinese. For a detailed discussion on the various usages of the term, see Yeh and Li, 2002). Radicals are crucial for reading development (Ho et al., 2003) and play an important role in character processing (Hsu et al., 2021; Yeh & Li, 2002). There are 201 main radicals, some with variants depending on their position in a character layout (State Language Affairs Commission, 2009). Neither MELD-SCH nor CLP covers this entire set.

Megastudies of visual word recognition often take the form of a lexical decision task. Although there are some disadvantages (Balota & Chumbley, 1984), one of the main advantages of this task is that the reaction times are straightforward to analyze. In megastudies, the design of pseudowords (or pseudocharacters) is of crucial importance. If participants, through implicit statistical learning, can identify properties that, with a high degree of likelihood, distinguish pseudocharacters from the character stimuli, they may start responding to whether or not a stimulus reflects those properties, rather than whether it is an existing character or not. The existing pseudocharacter-generating methods of arbitrarily combining subcomponents may face such a challenge.

In addition, understanding how pseudocharacters are processed enhances our understanding of how words are processed (Cassani et al., 2020; Hendrix & Sun, 2021). Although existing megastudies of Chinese characters which use a lexical decision task have taken great care in designing good pseudocharacters, MELD-SCH is the only existing megastudy that has published pseudocharacter latencies. However, 12 of its pseudocharacters are listed in the SUBTLEX-CH frequency corpus (Cai & Brysbaert, 2010), indicating that they might not be true pseudocharacters. Overall, we think that there is still room for improvement in this area.

In the current paper, we present the Simplified Chinese Lexicon Project (SCLP), which collects lexical decision responses for all 8105 characters in the *List of Commonly Used Standard Chinese Characters* and 4864 pseudocharacters carefully designed to reflect the properties and variations present in real characters.

## Method

### Participants

Participants were recruited at South China Normal University via electronic flyers distributed in a WeChat chat room specifically designed to announce experiments. The flyer informed potential participants that they could participate in a Chinese character recognition study that would take an average of 6 h to complete. Potential participants were informed that they would receive 200 RMB upon completion of the study, or 7 RMB per block if they decided not to continue the experiment along the way.

A total of 42 participants (27 female, 15 male) were recruited for the experiment. All participants took part in a pilot session in which four blocks of 20 stimuli were presented (12 characters and eight pseudocharacters per block). Participants were informed that they would not be able to continue with the experiment if they did not achieve the required score on two or more of the blocks in the test session (two participants did not continue) or on more than four blocks during the course of the experiment (three participants did not continue). In addition, eight participants dropped out of the experiment for personal reasons. Consequently, 29 participants (19 female, ten male) completed all blocks and responded to all stimuli. Their ages ranged from 18 to 24 (*M* = 20.31, *SD* = 1.97). To assess their proficiency in Chinese, participants’ scores on the Chinese Language in the National Higher Education Entrance Examination were provided as a reference (*M* = 114.07 out of 150, *SD* = 6.54).

### Stimuli

The stimuli consisted of 8105 characters and 4864 pseudocharacters. The characters were selected based on their inclusion in the *List of Commonly Used Standard Chinese Characters*, published by Language and Text Information Management Department (2013).

Following Keuleers and Balota (2015), an unequal proportion of characters (62.5%) and pseudocharacters (37.5%) was used to prevent participants from developing a bias towards negative responses. Considering a typical vocabulary size of 80% (estimated on the average accuracy in CLP & MELD-SCH) and a small percentage of positive responses to pseudocharacters, this ratio should lead an average participant to give about as many yes responses as no responses. In the current study, the large number of rare characters lead to an average accuracy of 61% for characters. Combined with the high correct rejection rate for pseudocharacters, this still resulted in a slight negative response bias.

**Fig. 1:**
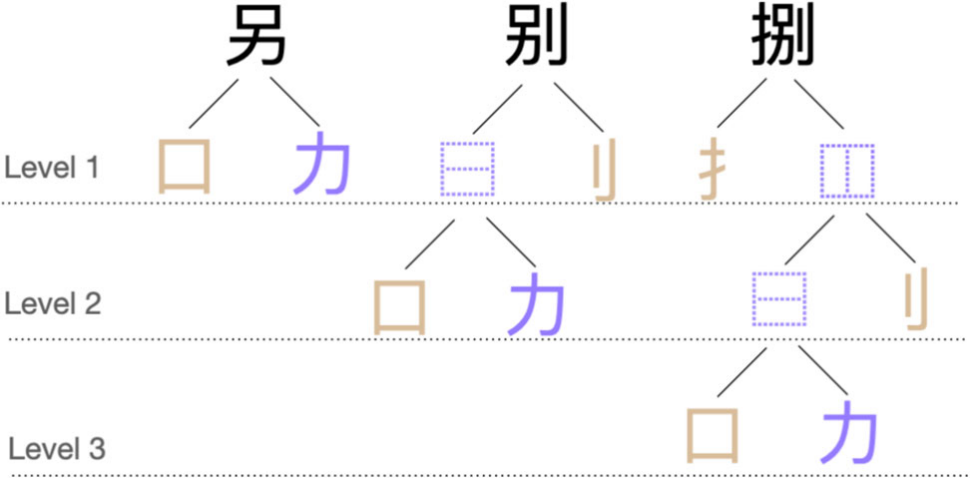
Three characters are shown to illustrate the decomposability of characters at different levels. At each level, the radicals of characters are printed in brown, and residual components are printed in purple. If the component is a character itself, a layout descriptor (collected from Unicode Consortium, 2023) is given as a placeholder describing the structure of its radical and residual component. For pseudocharacter generation, higher levels correspond to more intricate manipulations involving finer details and nested structures

**Fig. 2:**
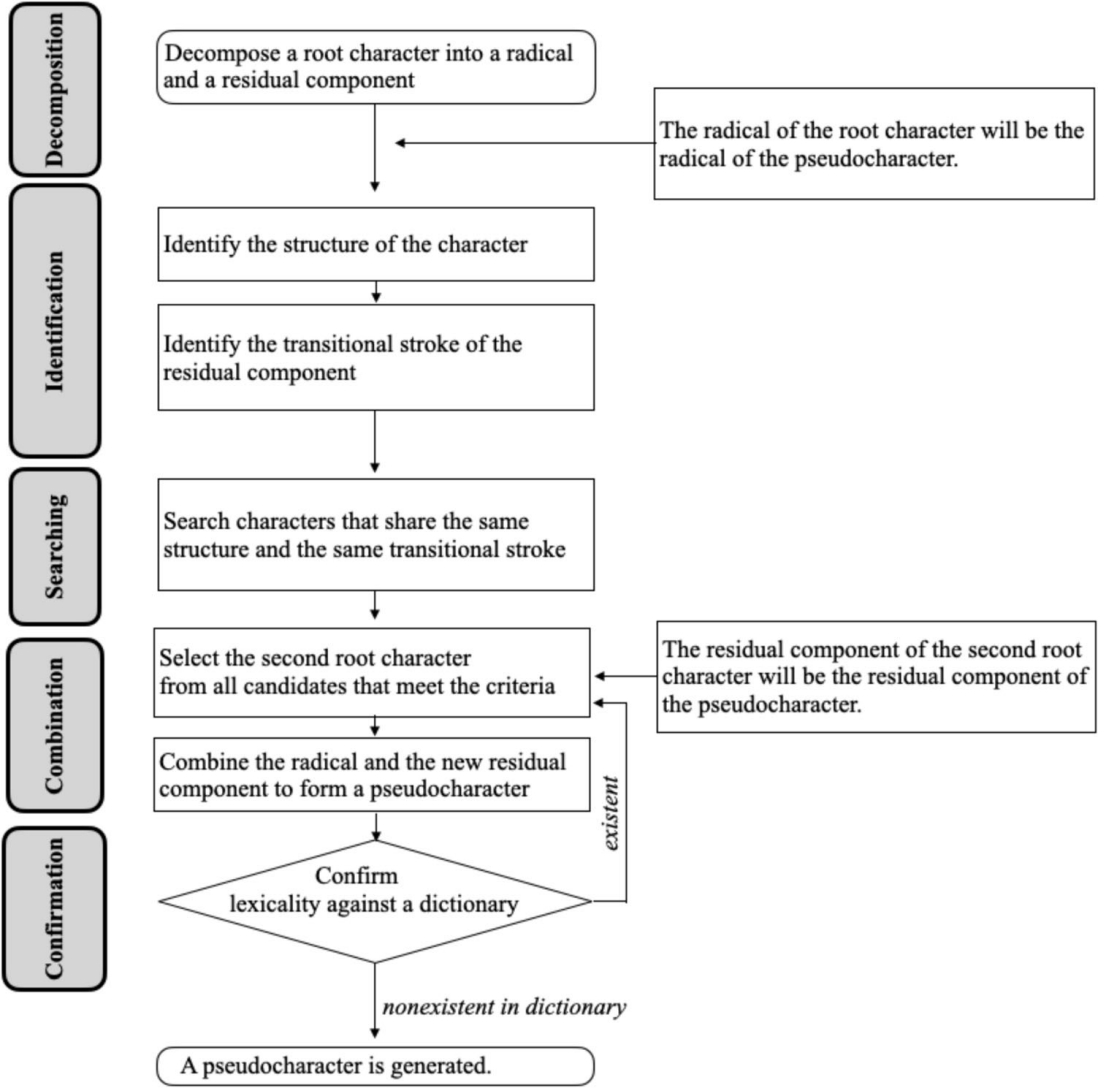
Example of how a pseudocharacter was generated. For level 1, there were five steps, including decomposition, identification, search, combination, and confirmation. Note that the selection of root characters for pseudocharacter generation was limited based on their structures and transitional strokes to increase character-likeness for a pseudocharacter. Methods for generating a pseudocharacter by manipulating subcomponents at level 2 or 3 were similar

Pseudocharacters were created based on the fact that characters can be hierarchically decomposed (see Fig. 1). Seen from a dichotomous perspective, characters, except those with integral structures, can be decomposed into a radical and a residual component. We define the level where manipulation of these subcomponents occurs as level 1. If the residual component itself is a decomposable character, then it can be further divided into a radical and a residual component, where the level is defined as level 2, and so forth. We set the maximum level to three. Such decomposability allows characters to be recomposed by hierarchically piecing together subcomponents. Thus, pseudocharacters can be generated through the exchange of subcomponents of two root characters at a certain level. While almost all existing methods (e.g., Sze et al., 2014) focus on swapping subcomponents at level 1, which has the advantage of retaining functions of subcomponents (e.g., possible phonetic information from residual components), deeper exploration into the hierarchical structure enables the creation at level 2 or beyond, resulting in distinctive pseudocharacters. However, we recognize that manipulation at deeper levels may produce outcomes with high visual complexity. To address this, constraints are imposed on levels 2 and 3. Specifically, stroke numbers of selected subcomponents are matched to render the resulting items plausible. The procedure outline is illustrated in Fig. 2. For a detailed explanation of generating pseudocharacters, see Wang et al. (2024).

We selected a total of 4864 pseudocharacters for the experiment, comprising 4603 at the first level of manipulation, 239 at the second level, and 22 at the third level. Each pseudocharacter’s lexical status was verified using the online Xinhua Dictionary (2024). We ensured that in the set of pseudocharacters, each radical occurred proportionally to its frequency in the set of characters. Similarly, the set of pseudocharacters matched the set of characters in the distribution of the number of strokes. Decomposition data for these characters were accessed from an online character stroke database (Hanzi Stroke Lookup, 2024).^1^

### Procedure

The entire task consisted of up to 12,969 trials, randomized for each participant before being divided into 26 blocks (the first 25 blocks of 500 trials and the last block of 469 trials). The experiment began with a test session where 80 trials divided into four blocks were presented in order to acquaint participants with the whole operation and the result was not recorded. At the end of each block, participants received a performance report, stating the accuracy rate for real characters and false rate for pseudocharacters. The feedback was followed by an instruction to take a 5-min break.^2^ For each day, participants were allowed to complete a maximum of four blocks of tasks, and the time they spent on the tasks should not exceed 2 h. Otherwise, they could stop early whenever they felt like it.

A trial consisted of a fixation sign and a stimulus (character or pseudocharacter). First, a fixation sign was presented in the center of the screen for 500 ms, followed 120 ms later by a stimulus. Participants pressed the button *j* if they thought the stimulus was a real character and *f* if it was a pseudocharacter. There was no time limit for the response, although participants were informed prior to the experiment that they should respond as quickly as possible with their best shot at accuracy. Note that participants were also explicitly instructed not to guess, but to use a strategy of familiarity to increase their scores. They were told: ”If you have ever seen or known these stimuli, there is a higher chance that it is a character. If you have not, it has a higher chance of not being a character.”

## Results

In this section, we present the findings of the current study. To provide descriptive statistics, we compare the current database with two existing databases in terms of their general properties. To establish the reliability of the dataset, we report split-half reliability and intraclass correlation coefficient (ICC). To illustrate the applications of the dataset, we report two virtual experiments investigating visual complexity, as well as neighborhood size and regularity. To demonstrate how the dataset can be used to offer new insights, we present an extensive analysis of how word frequency and subcomponent frequency play a role in (pseudo)character recognition.

### Descriptive measures

Only data from participants who made it to the end of the experiment were analyzed. As a first step, incorrect responses (26.42%) were removed. Following Keuleers et al. (2012), outliers in response times were identified using a non-parametric criterion, commonly used to identify outliers in box plots. For each participant and response class (word or non-word), data points that were below or above the first and third quartile by three times the interquartile range were removed (3.31%). In addition, data points with response times less than 200 ms were removed. Subsequently, standardized response times (zRTs) were computed per block, participant, and lexicality.

Table 2 shows descriptive statistics for the present study, compared to CLP (Sze et al., 2014) and MELD-SCH (Tsang et al., 2018). On average, response times were 50.69 ms lower than in MELD-SCH, but 202.2 ms higher than in CLP. Mean accuracy for word responses in the present study (0.61) was lower than in the other two databases. The high accuracy (0.94) for pseudocharacters shows that this was not due to higher noise in the response, but most likely due to the large number of low-frequency characters included in this dataset. This is supported by the fact that for characters shared between SCLP and CLP (*N* = 2497), the mean accuracy is close (0.94 for SCLP and 0.95 for CLP).

**Table 2:** Comparison between the current study (SCLP) and CLP (Sze et al., 2014) and MELD-SCH (Tsang et al., 2018)

	SCLP	CLP	MELD-SCH
Character			
*N*	8105	2500	1020
Frequency	2.43 (1.31)	3.15 (1.01)	2.46 (1.28)
RT	803.90 (173.65)	601.70 (80.23)	854.59 (161.72)
zRTs	.28 (.75)	-.19 (.35)	.05 (.53)
Accuracy	.61 (.38)	.95 (.10)	.82 (.23)
Pseudocharacter			
*N*	4864	2500	1020
RT	717.94 (72.62)	–	813.88 (76.10)
zRTs	.04 (.41)	–	-.09 (.25)
Accuracy	.94 (.09)	.95 (.02)	.91 (.09)

The frequency is the mean of the log10 character occurrences computed on the common characters between SUBTLEX-CH (Cai & Brysbaert, 2010) and the respective projects (*N* = 5298 for SCLP, *N* = 2482 for CLP, and *N* = 1020 for MELD-SCH). Mean and standard deviations for RT, zRTs, and accuracy are shown for both characters and pseudocharacters. For the CLP, only average accuracy was available for pseudocharacters

On average, participants in the current study and MELD-SCH responded 35–40% more slowly than participants in the CLP. This may stem from three factors beyond potential group characteristics like age or educational achievement. First, both the present study and MELD-SCH characters had a lower frequency than in CLP. Longer response times could therefore be attributed partly to the frequency effect. Second, the character / pseudocharacter discrimination may have been more difficult in the current study, which may have affected decision latencies. In CLP, pseudocharacters were generated by randomly combining one radical and one residual component of existing characters. As described earlier, we constructed pseudocharacters by decomposing characters into a finer hierarchical structure and matching the transitional frequencies between those components with those existing in real characters, making the pseudocharacters more character-like and the task more challenging. Both MELD-SCH and the current study show higher latencies for characters than for pseudocharacters, aligning with findings from the BLP (Keuleers et al., 2012). Finally, participants were instructed to prioritize familiarity when making decisions, which allows them to reject stimuli they had never encountered before but also potentially introduces an additional cognitive load, slowing down response times.

### Reliability

Following the methodology adopted by several other megastudies (Ferrand et al., 2010; Keuleers et al., 2012), we computed the split-half reliability, attenuated for length, for RT, zRTs, and accuracy. We randomly assigned participants to two groups, resulting in one group of 15 participants and another of 14. For both RT and zRTs, we calculated the mean response time and accuracy for each item per group. Then, we computed the correlation of the averaged response measures. After applying the Spearman-Brown formula, the split-half reliability estimates were .70 for raw response times, .75 for standardized response times, and .97 for accuracy. The increase in reliability observed from RT to zRTs shows that standardizing response times removes participant-specific biases, which tend to be associated with practice effects (Keuleers et al., 2010).

In addition, we computed two variants of the intraclass correlation coefficient (ICC; Shrout and Fleiss, 1979). The first ICC(2, 1) quantifies single-rater reliability. Based on the performance on a repeated lexical decision task, Diependaele et al. (2012) estimated this to be .5 for accuracy and .08 for RT. Our results are comparable: .18 for RT, .24 for zRTs, and .59 for accuracy. In nearly all circumstances, however, analyses are based on combined data from participants, for which the reliability estimate is ICC(2, k). In our data, this was .87 for RT, .9 for zRTs and .98 for accuracy. For comparison, the BLP (Keuleers et al., 2012) found ICC(2,k) to be .82 for RT, .87 for zRTs, and .96 for accuracy. Although this indicates that the reliability of this data is excellent overall (Cicchetti, 1994), we still advise caution when analyzing results of stimuli with low accuracy (Diependaele et al., 2012). A good practice is to select items with accuracy over a certain threshold (e.g., 2/3 of participants) and to use zRTs for analysis, when possible.

Table 3 shows correlations between response measures of the current study and those for shared stimuli with CLP and MELD-SCH. As expected, correlations for response times were slightly lower than correlations for standardized response times, consistent with other megastudies. For instance, when comparing the British Lexicon Project to the English Lexicon Project, Keuleers et al. (2012) found a correlation of .68 for RT and .77 for zRTs. For accuracy, there was a noticeably higher correlation with MELD-SCH (.87) than with CLP (.69). This is a bit surprising since there was almost no difference in accuracy between the CLP and the current study (.95 vs .94) for the shared characters that the correlation was based on.

**Table 3:** Correlations for response measures collected in the current project and in CLP and MELD-SCH, based on shared characters (*N* = 2497 for CLP, *N* = 982 for MELD-SCH)

	SCLPrt	SCLPzRTs	SCLPacc
CLPrt	.70	.70	-.53
CLPzRTs	.71	.73	-.56
CLPacc	-.54	-.56	.69
MELDrt	.71	.74	-.69
MELDzRTs	.73	.76	-.69
MELDacc	-.63	-.66	.87

Correlations between MELD-SCH and CLP are not shown because they only share 381 characters

### Virtual experiments

Megastudy data can be useful in understanding if and under which conditions previous experimental results replicate (Keuleers & Balota, 2015). In order to better understand the relation between the current study and earlier experimental data, we try to replicate findings from two virtual experiments investigating the effects of visual complexity, as measured by the number of strokes, and neighborhood size.

#### Visual complexity

Leong et al. , (1987, Experiment 2) investigated whether visual complexity, measured by number of strokes, affects character recognition. In a factorial lexical decision experiment, participants responded to a mixture of traditional Chinese characters, which had been categorized as having either low visual complexity (less than ten strokes) or high visual complexity (ten strokes or more). Characters in both groups were matched for character frequency. The results showed a significant effect of visual complexity: characters with fewer strokes were recognized about 100 ms (or 9 %) faster than characters with many strokes (see Table 4).

**Table 4:** Mean response times for characters with low and high visual complexity in this study and the original paper are shown

	Original Paper		SCLP	
	*N*Strokes	RT	*N*Strokes	RT
Low visual complexity	7.4	1045	7.2	649
High visual complexity	13.6	1139	11	718

We first replicated the study by matching the traditional characters to their simplified counterparts in our database. This provided RTs for the corresponding characters, but because simplification reduces stroke number, the average visual complexity was lower. Like in the original experiment, the main effect of stroke number was significant by participants $F_1$(1, 28) = 28.65, $p < .001$. This was also confirmed in a by-items analysis $F_2$(1, 38) = 7.77, $p < .01$. Interestingly, although participants in our experiment responded much faster than in the original experiment, the relative effect size remained similar (± 70 ms, or 11%).

**Fig. 3:**
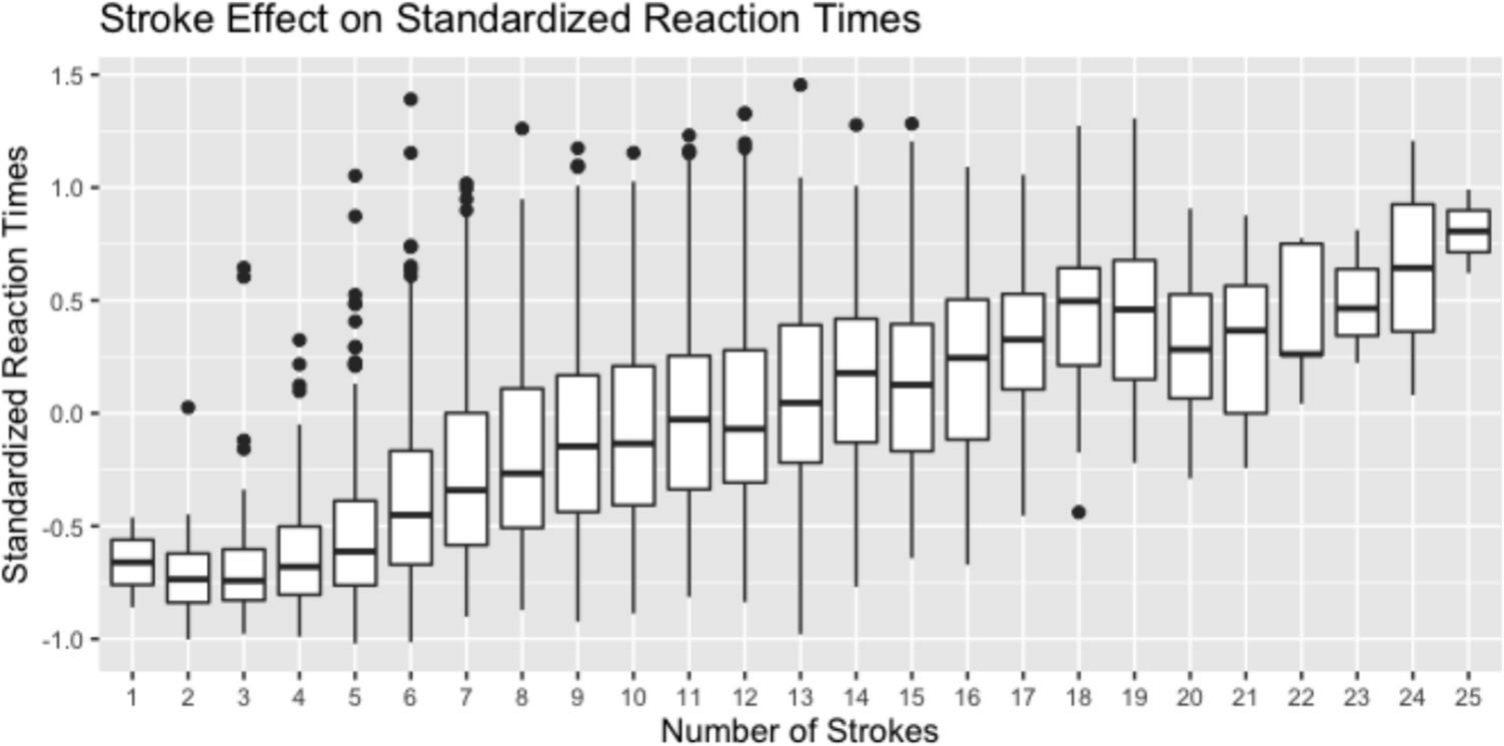
Box-and-whiskers plots of zRTs, by number of strokes, for characters with accuracy over .66 in the database

**Fig. 4:**
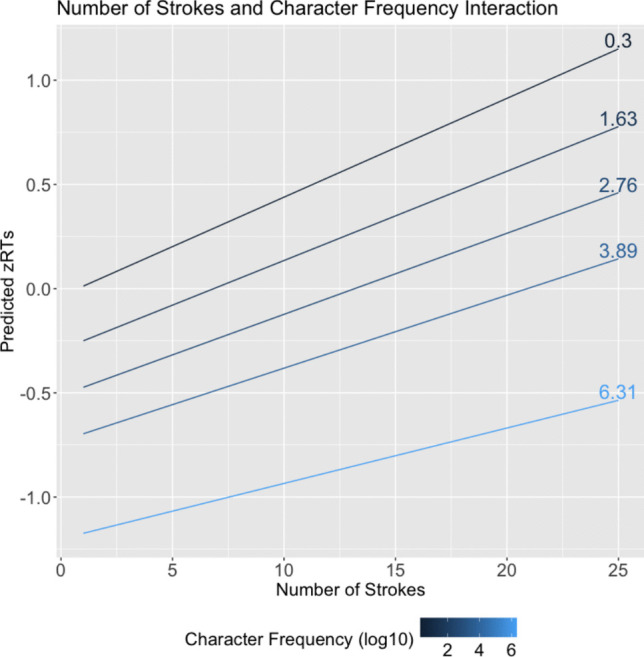
Effect plot illustrating the interaction between number of strokes and frequency (log-transformed) on predicted standardized response times (Predicted zRTs). Separate lines represent the effects estimated for the following frequency levels: minimal, one standard deviation below the mean, mean, one standard deviation above the mean, and maximum

Next, we tried to replicate the effect using the potential of our dataset by investigating the functional effect of numbers of strokes over all characters with an accuracy above 0.66 (*N* = 4582). Figure 3 shows box and whiskers plots of zRTs for characters, grouped by stroke number. The figure suggests that the effect of stroke number is concentrated in the range of 6–18 strokes. On average, characters with fewer than six strokes seem to be processed very quickly. For characters with more than 18 strokes, there does not seem to be a consistent penalty for increasing stroke number. Importantly, however, Fig. 3 does not control for frequency. This can be a significant factor because characters with fewer strokes tend to be more frequent. In a mixed effects analysis using subjects and items as random intercepts and stroke and frequency as fixed effects factors, we found that the effect of stroke number is largely independent of the effect of frequency (see Table 5 for analysis result and Fig. 4 for the effect plot).

#### Neighborhood size and regularity

The neighborhood size of a Chinese character is defined (e.g., Bi et al., 2009; Li et al., 2020) as the number of characters that share a phonetic component. For example, the character *ke1* ’a jade-like stone’ has the phonetic component  , which appears in 28 characters (e.g., *ke1* ’axe handle’, *he1* ’to exclaim’, and *ke3* ’rough’). Previous research on phonological neighborhood size has yielded contradictory results. Some studies (Li et al., 2011; Zhao et al., 2012, 7^th^ graders) found an inhibitory effect, others (Li et al., 2020, Experiment 3; Zhang and Jiang, 2008; Zhao et al., 2012, 3^rd^ graders) observed a facilitative effect, while other studies reported no effect (Li et al., 2020; Zhao et al., 2012, 5^th^ graders).

Regularity defines the mapping between the pronunciation of a character and its phonetic component, irrespective of tone. Previous findings suggest that regularity facilitated character naming, although studies often examine its interaction with other variables. Specifically, Fang et al. (1986) investigated regularity in a naming task, and found an effect due to consistency within regular characters but none specifically attributable to regularity itself. Lee et al. (2005) then added character frequency, and found longer naming latencies in low-frequency irregular characters compared with regular characters. Yum and Law (2019) reported an interaction effect between age of acquisition and regularity, with late-acquired regular characters eliciting attenuated N400 responses.

Zhou et al. (2021) investigated the interaction between neighborhood size and regularity in a lexical decision task. Their study, which used participants comparable to the ones in the present study, found that characters with a large neighborhood size were responded to significantly faster than those with a smaller neighborhood size (see Table 6). However, this effect was larger for irregular characters than for regular characters, yielding a significant interaction effect between neighborhood size and regularity. The main effect of regularity was not significant. According to the authors, the joint contribution of the many neighbors sharing similar but not identical pronunciations, leads to faster recognition for irregular characters; however, the same is not true for regular characters because they have *identical* pronunciations (excluding tone), leading to more difficulty integrating sound and appearance. This interpretation may need more evidence, especially given that the consistency (the relative value of neighbors that also have the same pronunciation) was the same for both groups.

**Table 5:** Effects for number of strokes (NStrokes), character frequency (CF), and their interaction on zRTs for characters with frequency entry in SUBTLEX-CH (Cai & Brysbaert, 2010) and an accuracy above 66% (*N* = 4394)

	*Estimates*	*SE*	*t*	*Chisq*	*p*
(Intercept)	.023	.039	.582	.339	.561
*N*Strokes	.049	.004	13.715	188.111	$<.001$
CF	-.194	.012	-16.124	259.968	$<.001$
*N*Strokes : CF	-.003	.001	-2.901	8.423	$<.01$

Character frequency was computed as log10 (x + 1) where x is the number of occurrences of a character in the corpus

All the 120 characters used by Zhou et al. (2021) were present in our database, but two had an accuracy below .66 (i.e., *ji2* ’level’ and *xing4* ’basic nature’). In addition, the original paper used the character ( *te4* ’particularly’) twice in one condition, resulting in 117 characters available for analysis. In contrast to the original study, our results show a main effect of regularity (*F*(1, 28) = 16.26, $p < .001$), an interaction effect between neighborhood size and regularity (*F*(1, 28) = 7.49, *p* = .01), but no main effect of neighborhood size itself (*F*(1, 28) = .30, *p* = .59). Using the same characters, in a linear mixed effects model (LMM) including items and subjects as random intercepts, we also found a main effect of regularity and an interaction between regularity and neighborhood size on response times (see Tables 7 and 8 for result analysis).

While we replicated the interaction effect between neighborhood size and regularity, the results cannot be interpreted in the same way. Specifically, in the original study responses to irregular characters with a large neighborhood size were 108 ms faster than responses to irregular characters with a small neighborhood size. In our study, the same responses were 24 ms slower. Taken together with other results in the literature, this suggests that the effects of neighborhood size and regularity in character recognition are unstable and may be highly dependent on experimental setup and extraneous factors.

**Table 6:** Mean response times for characters in four conditions manipulating neighborhood size and regularity are shown

		Regular		Irregular	
		Large NS	Small NS	Large NS	Small NS
RT	Original	581(110)	607(87)	570(95)	678(95)
	SCLP	647(58)	673(66)	693(63)	669(59)

**Table 7:** Effects for neighborhood size (NS) and regularity on response times

	*Estimates*	*SE*	*t*
(Intercept)	691.83	16.07	43.055
SmallNS	-21.89	15.94	-1.374
Regular	-46.05	16.05	-2.869
SmallNS : Regular	48.32	22.40	2.157

### Character frequency and subcomponent frequency in character recognition

As discussed in the previous section, most methods define a character’s neighborhood by similarity to its phonetic component, while some methods take into account semantic components (e.g., Zhang and Jiang, 2008). However, the interaction between the frequency of subcomponents remains unexplored, which also applies to non-phonograms. In this section, we evaluate the effects of character frequency and the frequency of subcomponents (i.e., radical and residual components) on lexical decision response latencies using linear mixed effects models.

#### Independent Variables

**Fig. 5:**
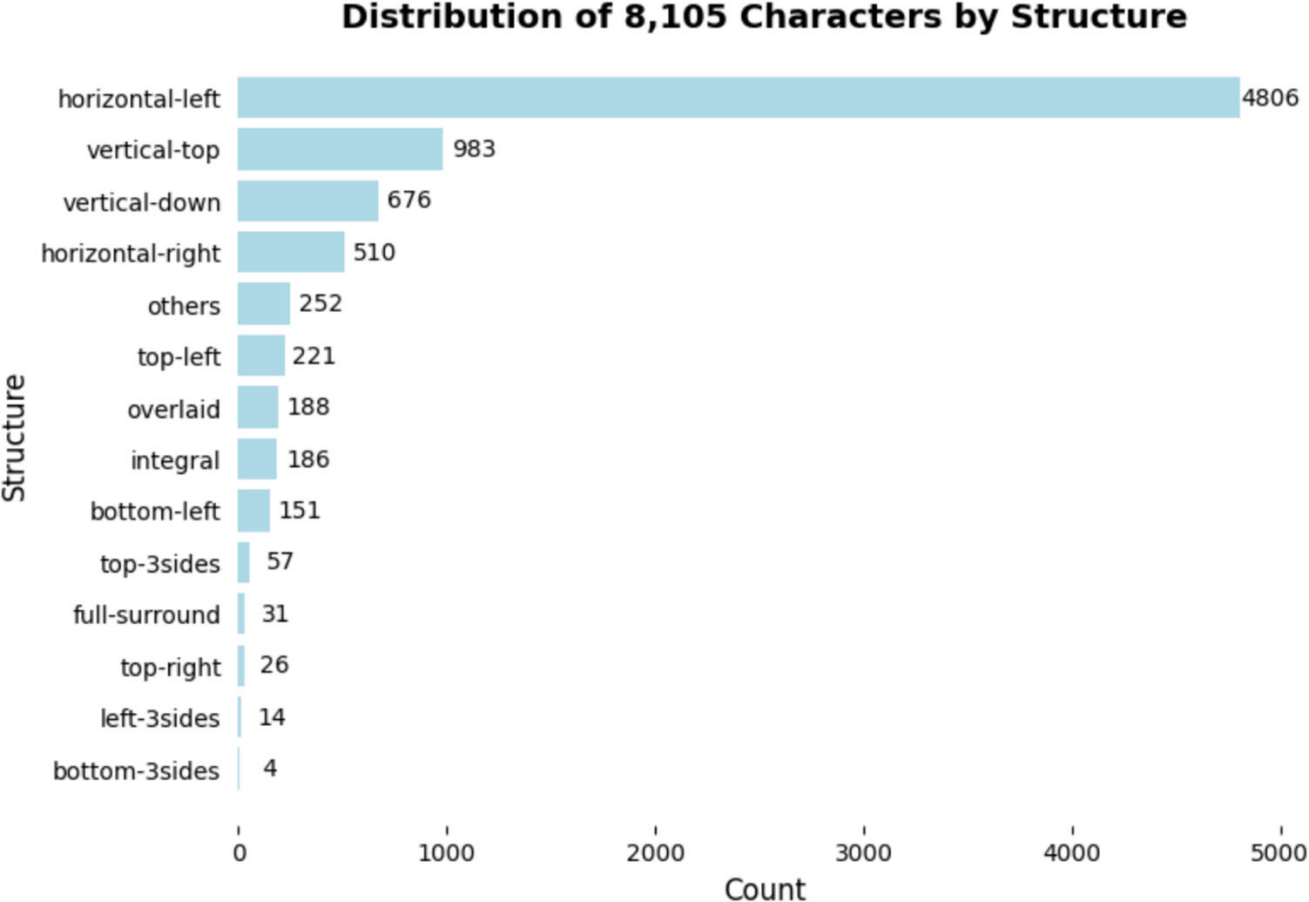
The number of characters by structure counted from the whole set of 8105 is shown. There are six basic types of structures: horizontal, vertical, full-surround, semi-surround, overlaid and singular. Since subcomponents can appear in different positions, we indicate the position of the radicals in the names of specific structures. For example, horizontal-left denotes horizontal characters with the radical on the left, and the residual component is located on the right. Note that the following indicates the position of radicals for semi-surround characters: top-left, bottom-left, top-3sides, top-right, left-3sides, bottom-3sides. *Others* are semi-surround characters where the position of radicals is not categorized

For the present analysis, we define character frequency as the number of occurrences of a character, collected from SUBTLEX-CH (Cai & Brysbaert, 2010). Radical frequency, also known as radical combinability (Feldman & Siok, 1999; Hsiao et al., 2007; Taft & Zhu, 1997), is the number of characters that contain the same radical. While this procedure seems straightforward, its operationalization requires some attention. First, glyph variation must be taken into account when counting radical frequency. Some radicals have variants that are orthographically distinct. For example, the radical *shui3* ‘water’ retains its original form in *miao3* ‘flood’, but transforms into  when used as a building block in *he2* ‘river’. In this case, the frequency of radicals is counted separately. Second, the position of a radical must be considered, as it has been shown to influence character recognition (Liu et al., 2022; Taft & Zhu, 1997; Yum et al., 2015). To illustrate the varying structures in which a radical can appear across characters, consider the radical  . It occurs in several characters with different layouts: *zhi3* ‘only’ (vertical layout), *wu2* ‘I’ (vertical layout), *ye4* ‘leaf’ (horizontal layout), *tong2* ‘same’ (semi-surround layout), *ke3* ‘okay’ (semi-surround layout). Figure 5 illustrates the distribution of layouts for all characters in the present study. To clarify the contributions of position, we consider two types of radical frequencies: position-dependent and position-independent. For the same reason, residual component frequency (i.e., the number of times a residual component occurs in characters) has two versions: position-dependent and position-independent, while there is no glyph variation of residual components.

**Table 8:** Analysis of variance for the linear mixed effects model investigating neighborhood size (NS) and regularity on response times

	*Chisq*	*p*
(Intercept)	1853.72	$<.001$
NS	1.888	.169
Regularity	8.229	$<.01$
NS : Regularity	4.654	$<.05$

Both radical frequency and residual component frequency were type frequency counted on the basis of the complete data set of 8,105 characters. To address data skewness, character frequency and radical frequency were log-transformed using log10 (frequency measure + 1), while raw residual component frequency remained unaltered. We built two models involving either position-independent subcomponent frequency or position-dependent subcomponent frequency, alongside character frequency in both. For simplicity, these two models are hereafter referred to as the *position-independent* model and the *position-dependent* model.

#### Data description

For analysis, we selected the characters from our study that consisted of a radical and a residual component, had an accuracy higher than .66, and had a frequency attested in SUBTLEX-CH. Variable correlations for these 4225 characters are shown in Table 9.

#### Model selection

Following Tremblay and Tucker (2011), we employed an iterative fitting process for model selection. The entire process comprised the following steps. (1) An initial model was built. (2) Outliers were identified and removed if their residuals were outside the scope of ±2.5 standard deviations. (3) Fixed effects factors were excluded via back-fitting. (4) Random slopes of fixed effects factors were added via forward-fitting. 4) Fixed effects factors were retested via back-fitting.

**Table 9:** Correlation between standardized response times and independent variables for characters (*N* = 4225) used in the initial models

	zRTs	CF	$RF_{pi}$	$RF_{pd}$	$ResF_{pi}$	$ResF_{pd}$
zRTs	–	-.399	.119	.150	.031	.089
CF		–	-.146	-.172	-.054	-.118
$RF_{pi}$			–	.911	.113	.271
$RF_{pd}$				–	.167	.322
$ResF_{pi}$					–	.696
$ResF_{pd}$						–

zRTs: standardized response times; CF: character frequency; $RF_{pi}$: position-independent radical frequency; $RF_{pd}$: position-dependent radical frequency; $ResF_{pi}$: position-independent residual component frequency; $ResF_{pd}$: position-dependent residual component frequency

We first fit a model with fixed effects factors, including the three independent variables, three two-way interactions and one three-way interaction, and random intercepts by subject and by item. Next, outliers were identified and removed if their residuals were outside the scope of ±2.5 standard deviations and the model was refitted again with the filtered data. The fixed effects factors were then treated using a back-fitting mechanism. The exclusion of terms proceeded in a top-down fashion, starting with a highest-order interaction, descending to lower-order interactions, and ending when all main effects were tested. To determine whether to retain a factor, we constructed a new model excluding the term and compared it with the original using a log-likelihood ratio test (LLRT), retaining the model with a better fit. The tests of multiple two-way interactions and main fixed effects factors were performed in order of increasing *t*-value. According to the marginality principle (Venables, 2000), if a term was part of a higher-interaction term that had a significant effect, it was retained even if its exclusion improved model fit. Next, we examined random slopes through a forward-fitting process, testing random slopes of survived main fixed effects factors in order of increasing *t*-values. Both by-subject and by-item random slopes were tested, with any leading to non-convergence dropped to maintain model parsimony (Barr et al., 2013). Last, fixed effects factors were retested by back-fitting. After taking all the steps, we kept the model maximal (Barr et al., 2013), while ensuring the robustness of the result against extreme values (Tremblay & Tucker, 2011).

**Table 10:** Effects for log-transformed character frequency (logCF), log-transformed radical frequency (logRF) and residual component frequency (ResF) on zRTs for the position-independent model and the position-dependent model

	*Estimates*	*SE*	*t*	*Chisq*	*p*
Position-independent model					
(Intercept)	.251	.072	3.470	12.044	$<001$
logCF	-.227	.022	-10.210	104.250	$<.001$
logRF	.124	.034	3.603	12.980	$<.001$
ResF	.021	.007	3.080	9.485	$<.01$
logCF : logRF	-.012	.011	-1.134	1.285	.257
logCF : ResF	-.005	.002	-2.528	6.389	$<.05$
logRF : ResF	-.015	.003	-4.321	18.669	$<.001$
logCF : logRF : ResF	.004	.001	3.692	13.633	$<.001$
Position-dependent model					
(Intercept)	.227	.054	4.196	17.604	$<.001$
logCF	-.228	.016	-13.911	193.503	$<.001$
logRF	.134	.026	5.095	25.959	$<.001$
ResF	.030	.008	3.556	12.642	$<.001$
logCF : logRF	-.011	.008	-1.277	1.631	.202
logCF : ResF	-.004	.003	-1.548	2.396	.122
logRF : ResF	-.021	.004	-4.895	23.965	$<.001$
logCF : logRF : ResF	.004	.001	3.078	9.474	$<.01$

#### Data Analysis and visualization software

We conducted the analysis using R (Ihaka & Gentleman, 1996). We used the *lme4* package (Bates et al., 2015) to run the linear mixed models and estimate both fixed and random effects. The programs from the package *LMERConvenienceFunctions* (Tremblay et al., 2020), including *bfFixefLMER_F.fnc* and *ffRanefLMER.fnc*, were used to select models. Model significance was determined using *Anova* from the *car* (Fox & Weisberg, 2019b) package. Other packages for data analysis and visualization included *effects* (Fox & Weisberg, 2019a) and *ggplot2* (Wickham, 2016).

#### Results

**Fig. 6:**
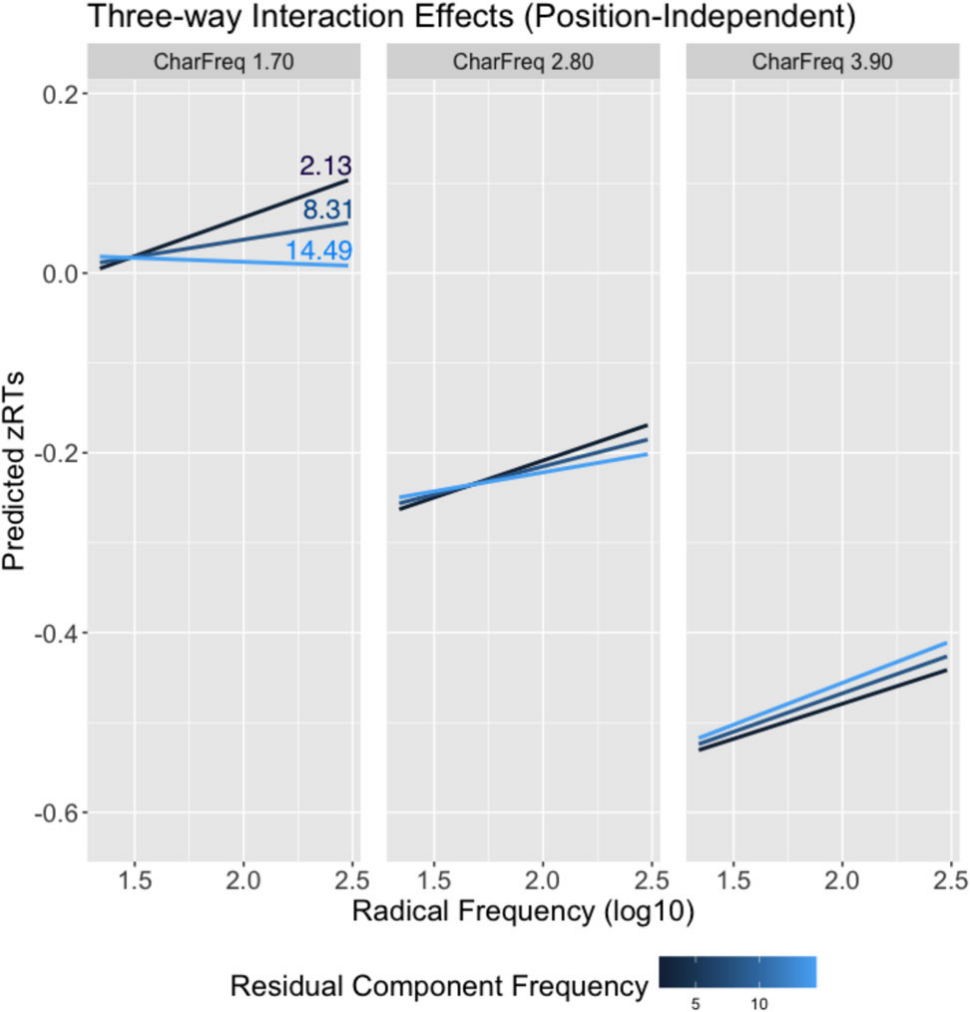
Effect plot illustrating the three-way interaction among character frequency (CharFreq), radical frequency, and residual component frequency in the position-independent model

**Fig. 7:**
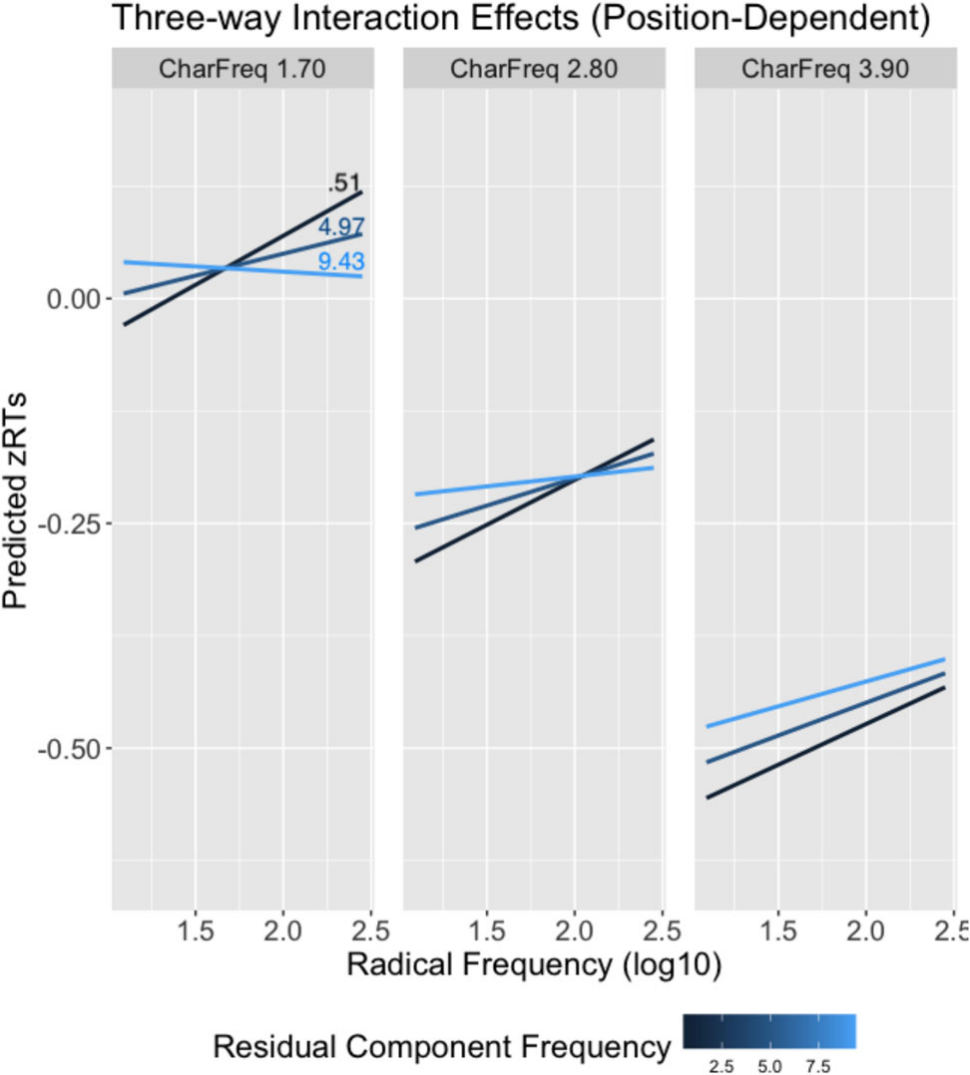
Effect plot illustrating the three-way interaction among character frequency (CharFreq), radical frequency, and residual component frequency in the position-dependent model

For both models, the fixed effects factor that did not improve model fit (i.e., residual component frequency) was still retained, as it was part of higher-order interactions. In addition, both models included by-item and by-subject random intercepts, along with random slopes for character frequency and radical frequency. Table 10 shows the results for the fixed effects factors. Figures 6 and 7 show effect plots of the three-way interaction.

#### Discussion

To our knowledge, this is the first attempt to study the interaction of subcomponent frequencies with character frequency by using characters of all types and structures. There are some highlights of the result: (1) We demonstrate a character frequency effect. (2) We report that the mechanisms underlying recognizing high-frequency characters and low- and medium-frequency characters are different. (3) The interaction of subcomponent frequencies appears only for low- and medium-frequency characters. (4) Taking position into account, we can see a more pronounced effect of residual component frequency in high-frequency characters.

**Fig. 8:**
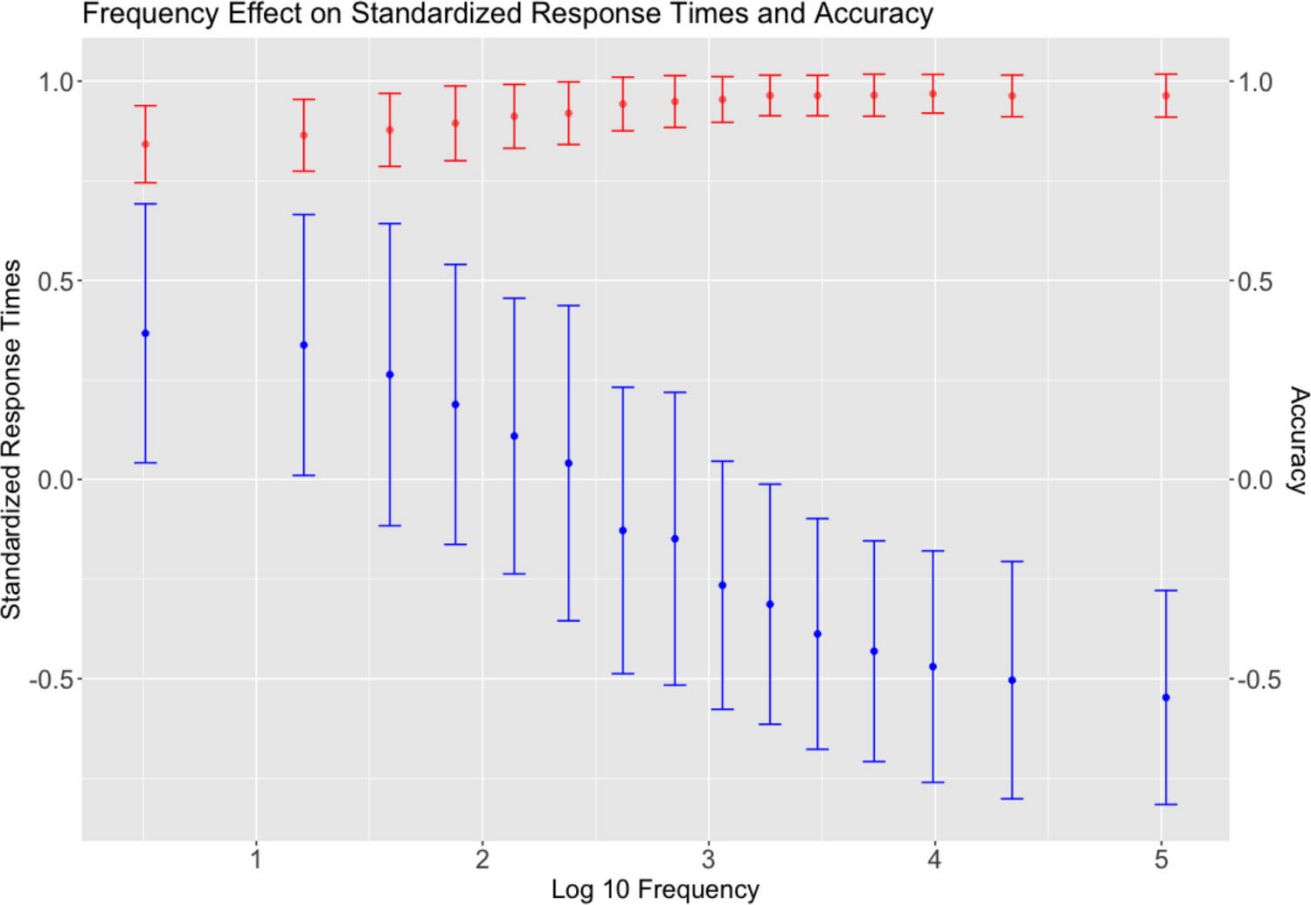
Frequency effect on standard response times is shown in blue and that on accuracy is shown in red. Frequency measures, i.e., the log 10 of the number of character occurrences, were retrieved from SUBTLEX-CH database (Cai & Brysbaert, 2010). Common characters with accuracy over .66 (*N* = 4394) in the present study were used and grouped with a size of 300 in each bin, except for the last bin of 94 items. Each dot represents the average frequency of a particular group on the *x*-axis, and the standardized response times and accuracy on the *y*-axis. A line was added to each point to indicate one standard deviation from the mean

The frequency effect is indeed one of the most robust effects found in word recognition. It suggests that the more frequently a word is encountered, the more accurately and easily it is recognized (Brysbaert et al., 2018; Forster & Chambers, 1973; Hronskỳ & Keuleers, 2021; Keuleers et al., 2010; Monsell et al., 1989). This has been confirmed across tasks and languages (see Brysbaert et al., 2018 for a detailed update). The current paper also reports a strong frequency effect. Figure 8 illustrates a clear decline in response times and an increase in accuracy as a function of character frequency.

The interaction between radical frequency and residual frequency depends on character frequency. Low- and medium-frequency characters have similar patterns. For low-frequency characters, characters with low radical frequency and low residual component frequency are recognized faster, while characters with high radical frequency and high residual component frequency are recognized faster. For medium-frequency characters, the interaction still holds but participants increasingly lean towards using radicals to make decisions, revealing a slight inhibitory radical frequency effect. For highly frequent characters, the residual component frequency effect approaches zero. Despite this, radicals continue to have an inhibitory effect.

The different patterns between high-frequency characters and the remaining may suggest that language users rely on different mechanisms when processing common and less common characters. According to the Dual Route Cascaded (DRC) model (Coltheart et al., 2001), when naming a common English word, the phonology of the word is directly activated, whereas when processing low- and medium-frequency word, the sub-lexical route which contains rules of grapheme-to-phoneme conversion is activated. Although we use a lexical decision task, there also seem to be two routes in character recognition: one where the whole character is activated, with only one subcomponent (i.e., radical) inhibiting such activation (for high-frequency characters) and one where both subcomponents interact and cooperate to help access a target character (for low- and medium-frequency characters). For high-frequency characters, direct activation results possibly from long-term exposure of a character as a whole, which leads to easier access, and enhanced memory with easier information retrieval. The radical also seems to impact the process. The more characters share the subcomponents, the longer it takes to activate the target. This is consistent with the interactive activation and competition model of McClelland and Rumelhart (1981), where activating a subcomponent would activate the pool of neighbors sharing it, possibly causing inhibition. Yet, such neighborhood competition does not seem to be present for characters sharing residual components. While the reason for the absence of this effect is not entirely clear, it is important to note that there are seven times more unique residual components than unique radicals. Thus, on average, a character will have fewer neighbors sharing its residual component than sharing its radical. Inhibition originating from neighbors sharing the residual component could therefore also be less pronounced.

For less frequent characters, however, the analysis becomes more complex and participants rely on familiarity with both subcomponents to process a character. The degree to which a low-frequency character ”resembles” a real character increases when both subcomponents are more common. In other words, when presented with a character that is not encountered often, participants start the analytic process with an efficient strategy: to see if he/she is familiar with both subcomponents. Another possibility may be related to word response bias (i.e., participants accept a low-frequency character with familiar subcomponents without recognizing it). The nature of lexical decision tasks is that they are fast binary decision tasks, and under time and accuracy constraints, participants tend to make a word response (Diependaele et al., 2012; Piercey, 2005). In this case, the response bias may become stronger for lower-frequency characters.

When we include the position of the subcomponents, we see similar patterns. This similarity might come from the strong correlation between subcomponent frequency in both conditions, measuring at .911 for radical frequency and .696 for residual component frequency. In addition, this indicates a relatively fixed position for radicals, while the position of a residual is more diverse. For the position-dependent model, residual component frequency scales down from that used in the position-independent model (mean residual component frequency has a gap of 35% between the two conditions), possibly leading to the minor difference seen in the high-frequency characters.

To examine these effects in the presence of extraneous variables, we retrieved data for the perceived number of meanings from Chen et al. (2024), for the number of word formations, concreteness, familiarity, and imageability from Liu et al. (2007), and for the number of strokes from our own count. This resulted in a subset of 2,202 characters with data available for all variables.

An initial correlation analysis showed that some variables were moderately or highly correlated. For example, character frequency had a moderate to strong Spearman correlation with familiarity (.42), the perceived number of meanings (.56), and the number of word formations (.75).

Before fitting LMMs, we applied transformations to improve the normality of the independent variables. Consistent with the method used in Hendrix and Sun (2021), we applied transformations suggested by the Box-Cox method: log(x+1) for character frequency, radical frequency, and number of word formations; sqrt(x) for number of strokes; x$^{2}$ for familiarity and imageability; and 1/x$^{2}$ for perceived number of meanings. In addition, to address concerns of potential multicollinearity, following Hendrix and Sun (2021), we applied a principal component analysis (PCA) on the transformed predictors, using varimax rotation. As a result, each predictor was clearly represented by a corresponding rotated component.

Then, we ran two linear mixed-effects models, with the same model selection procedure as described above. The results show that the main effects of character frequency and subcomponent frequencies were qualitatively similar to the effects reported in the previous analyses. However, the effect of residual component frequency was no longer observed for the position-dependent model. Additionally, two significant interactions between character frequency and subcomponent frequency measures were found in the position-dependent model. In the position-independent model, some interaction terms were no longer significant, but these interaction effects were also not very pronounced in the original model. In summary, while the inclusion of extraneous variables introduced discrepancies for some interaction terms, the overall pattern of results remained consistent.

There are two aspects to consider for improvement. First, only type frequency is used for subcomponents, not token frequency. Although different frequency effects may be processed in a way that converges over time, they have clear distinctions and may be active at different time spans (Pfänder & Behrens, 2016). Second, there is a potential confounding effect between frequencies and functions (e.g., possible phonological cues from residual components) in the analysis of subcomponents Liu et al. (2022). However, these functions are not the main focus of this paper. Future studies could explore the interplay between frequency and function in character recognition, investigating whether the reported effect of subcomponent frequency is the effect of semantic and/or phonological cues in disguise, or vice versa, or whether there are additive effects.

### Radical frequency and residual component frequency for pseudocharacter recognition

Since subcomponent frequency measures affect character recognition, it is interesting to see whether they also play a role in pseudocharacter recognition. To address this, we ran linear mixed effects models to test the effects of subcomponent frequency on response measures for pseudocharacters in the present study.

**Table 11:** Correlation between dependent variables and independent variables for pseudocharacters (*N* = 4750) used in the initial models

	zRTs	$RF_{pi}$	$RF_{pd}$	$ResF_{pi}$	$ResF_{pd}$
zRTs	−	.074	.096	.162	.203
$RF_{pi}$		−	.935	-.176	-.051
$RF_{pd}$			−	-.144	-.018
$ResF_{pi}$				−	.778
$ResF_{pd}$					−

#### Independent variables, model selection, and materials

We computed position-dependent and position-independent versions of radical frequency and residual component frequency for pseudocharacters. Radical frequency was log-transformed using log10 (frequency measure + 1) due to skewness, while raw residual component frequency was used. For model building, we followed the steps explained in detail in the study above. A total of 4750 pseudocharacters whose accuracy was higher than 66% were used to fit the initial models. Table 11 shows the correlations of the variables.

#### Results

Both models had by-item intercepts. Results in Table 12 show significant main effects of radical frequency and residual component frequency, and significant interactions for the position-independent model. Meanwhile, the position-dependent model demonstrates significant radical frequency effect and interaction effect.

**Table 12:** Effects for radical frequency (logRF) and residual component frequency (ResF) on zRTs for the position-independent model and the position-dependent model for pseudocharacters

	*Estimates*	*SE*	*t*	*Chisq*	*p*
Position-independent Model					
(Intercept)	-.390	.025	-15.320	234.687	$<.001$
logRF	.076	.012	6.247	39.030	$<.001$
ResF	-.008	.003	-2.636	6.949	$<.01$
logRF : ResF	.015	.015	10.155	103.124	$<.001$
Position-dependent Model					
(Intercept)	-.376	.018	-20.840	434.298	$<.001$
logRF	.068	.009	7.447	55.459	$<.001$
ResF	-.006	.004	-1.666	2.777	.096
logRF : ResF	.022	.002	11.397	129.900	$<.001$

**Fig. 9:**
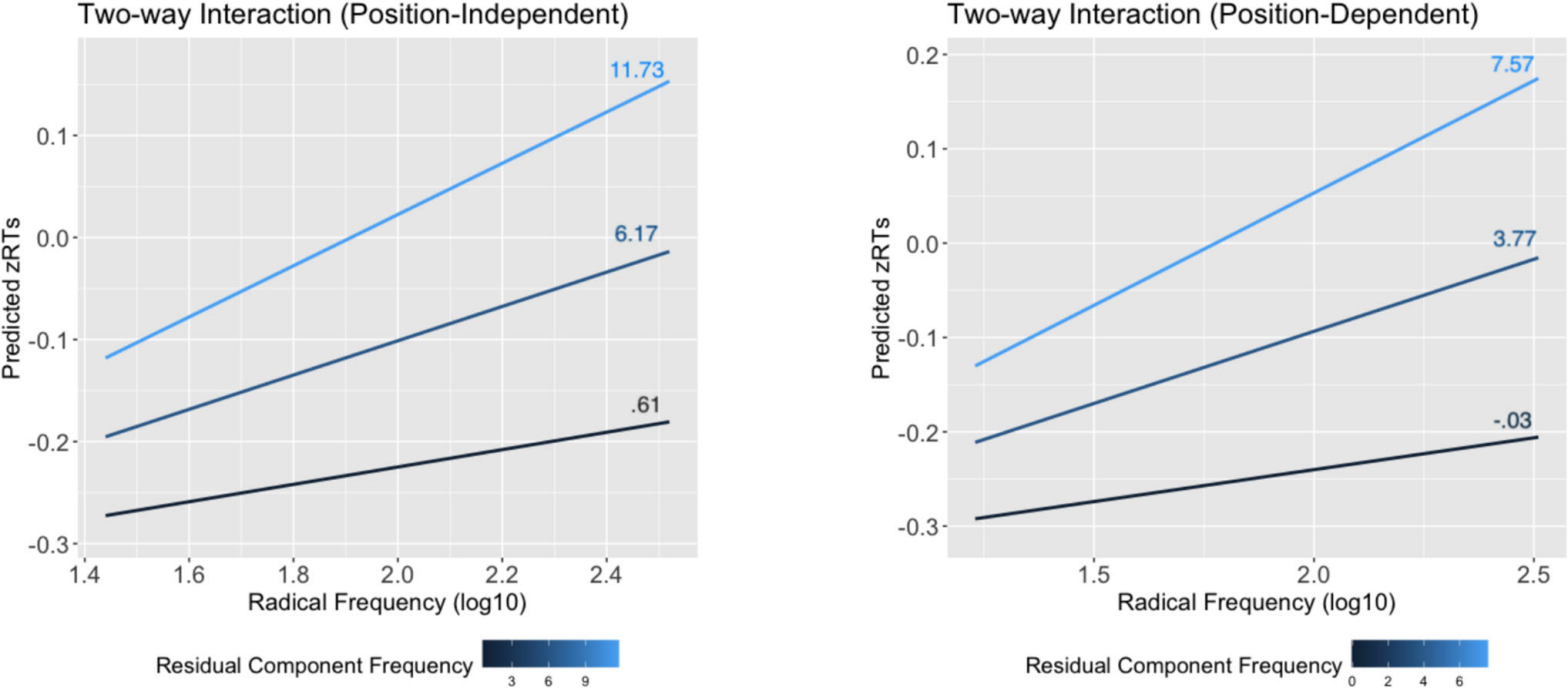
Effect plots of two-way interactions between radical frequency (log-transformed) and residual component frequency in pseudocharacters

#### Discussion

Table 12 and Fig. 9 show that, overall, position-dependent or not, there is a discernible inhibitory effect of radical frequency on pseudocharacter rejection latency. While residual component frequency appears to have a facilitative effect in the position-independent model, its impact is minimal, with no significant effect in the position-dependent model. The most striking observation resulting from the analysis is that residual component frequency and radical frequency interact in a very specific way and that when both are high, they combine to strongly delay the decision process. In other words, participants find it hard to reject pseudocharacters with both high residual component frequency and high radical frequency.

This suggests that when participants are faced with a decision to identify a stimulus as a character or not, they tend to look at whether *any* of the stimulus’s components are unfamiliar. If that is the case, the stimulus can be more easily identified as a pseudocharacter. Also, the combination of higher radical frequency and higher residual frequency may simply mean that more characters are activated at the same time. The large pool of characters that contain these subcomponents compete with each other, increasing the time it takes to rule out the possibility that the stimulus is a real character.

The task seems to be further complicated by the functions of subcomponents. Radicals often convey semantic knowledge about a character, activating common semantic associations shared by all characters containing the same radical. The larger the pool, the more challenging it is to reject a pseudocharacter, especially when they incorporates a residual component that appears *pronounceable* due to its inherent pronunciation from root characters. Interestingly, all pseudocharacters with relatively low accuracy (less than .67%) featured residual components that are phonetic components of real characters. In a follow-up interview at the end of the experiment, one participant expressed surprise upon learning that a pseudocharacter did not exist, as the participant was ready to describe its meaning and pronounce it. Future research could further explore the functions of subcomponents, but these aspects are beyond the scope of this paper.

## General discussion and conclusion

In this paper, we presented the Simplified Chinese Lexicon Project (SCLP), which collects lexical decision data for the full set of simplified Chinese characters that are attested in the *List of Commonly Used Standard Chinese Characters*. Our analysis shows that the internal reliability of the collected data is comparable to that of existing megastudies. The SCLP adds to the body of work established by the two earlier character recognition megastudies (CLP and MELD-SCH) and gives researchers the ability to compare results across several studies.

The data collected in the SCLP have the potential to contribute to our understanding of Chinese character processing. As we have shown, the data can be used to perform virtual factorial experiments or to understand the continuous effects of variables on character processing. In the investigations presented here, we showed that there is a robust effect of stroke numbers across all characters, that character processing is affected by an intricate interplay between neighborhood size and regularity, and that interactions between character frequency and subcomponent frequencies affect character recognition. In this context, we think that the way regularity is defined for Chinese characters can be improved. Current definitions of regularity are either binary (e.g., Fang et al., 1986) or confined to small-scale categorizations (e.g., Liu and Zhang, 2020). A more sophisticated approach to defining regularity would be to use more fine-grained distance metrics, like Levenshtein Distance, to measure the relative distance between the pronunciation (pinyin) of the subcomponents of a character and its pronunciation as a whole. This would allow researchers to study the effect of phonological mapping on a continuous scale using the entire character set provided in this study.

For the SCLP, we carefully designed and selected pseudocharacters to avoid implicit learning (Reber, 1989). Instead of arbitrarily combining subcomponents, we controlled for the transitional frequency of strokes between subcomponents. We also ensured that the range of visual complexity present in the set of characters was matched in the set of pseudocharacters. Our set of pseudocharacters also reflects, to a large extent, the proportions with which specific radicals and residual components occur in real characters. The large variation in pseudocharacters could facilitate future research on the role of subcomponents in Chinese character recognition. The pseudocharacter generation method used for the SCLP could also be extended to studies using multi-character words. Typically, multi-character pseudowords are generated by combining existing characters, which is efficient in terms of material preparation, but ignores that characters themselves have compositionality. The findings of the present study suggest that investigating the processing of pseudowords comprised of complex pseudocharacters could further elucidate the role of subcomponent properties in Chinese word recognition.

Despite being a key feature and advantage of this study, the large number of low-frequency characters included in the SCLP requires some caution. For instance, analysis of megastudy data often requires stimuli that have been correctly identified by at least two-thirds of the participants. In the case of the SCLP, only slightly more than half of the characters fit this criterion. On the other hand, the accuracy data collected here can also be used as an estimate of character knowledge. This can have applications in the design of vocabulary testing, with decreasing accuracy corresponding to increasing difficulty.

Because the majority of Chinese words consist of two characters, researchers should be cautious about generalizing findings based on the SCLP to Chinese word recognition in general. To provide a more detailed picture of visual word recognition in Chinese, findings based on the SCLP data should be contrasted to those from two-character recognition megastudies. Future work could also investigate the differential dynamics of character frequency and word frequency in word processing.

Finally, the lexical decision task’s simplicity does not detract from its limitations. Response times are not direct reflections of lexical access but also include post-recognition decision components (Perea et al., 2002; Polich & Donchin, 1988; Seidenberg et al., 1984). It is always advisable to contrast results found using lexical decisions with those found using tasks without an explicit decision component, such as fixation duration measures extracted from eye-movement studies. In addition, the statistical power of the database for virtual experiments should be evaluated on the same level as within-subject experiments and that, therefore, the database likely lacks power to find very small effects, i.e., on the order of $<20$ms, unless the comparison involves very large amount of stimuli (see Brysbaert and Stevens, 2018; Keuleers et al., 2010).

Raw results and trial-level data (TrialsSCLP.csv) can be accessed at https://osf.io/hgves/. The trial-level data file comprises a 376,101 $\times$ 7 matrix, with the following columns:

- **item**: a character or pseudocharacter. A pseudocharacter is represented by its two root characters.
- **subject**: subject identification.
- **lexicality**: indicating character or pseudocharacter status.
- **level**: 1, 2, or 3. In the context of pseudocharacter generation, this denotes the hierarchical level at which subcomponents are manipulated. For characters, the value is fixed at 1.
- **accuracy**: binary accuracy state.
- **rt**: raw response times.
- **zscore**: standardized response times, recommended for data analysis.

Researchers can also download images used in the experiment (available at https://github.com/yixiawang/SCLP-Supplementary-Materials), which are organized in two folders: one containing character images and the other containing pseudocharacter images. Each image follows a structured naming convention, consisting of the item, its radical, and the abbreviated layout. For example,  -hl and  -hl.

## Data Availability

The trial-level data are available at https://osf.io/hgves/, while images of stimuli used in the experiment are accessible at https://github.com/yixiawang/SCLP-Supplementary-Materials.

## Footnotes

1 Two pseudocharacters were later identified as real characters *jue1* ’to protrude’ and *jue2* ’a type of digging tool’. We traced this to the stroke data for the character  being incorrectly stored in the stroke database.

2 Programming was done using JsPsych (De Leeuw, 2015).

## Code availability

Code used in analysis is available at https://github.com/yixiawang/SCLP.

## Declarations

### Open Practices Statements

Materials and analysis code are available at https://osf.io/hgves/, https://github.com/yixiawang/SCLP, and https://github.com/yixiawang/SCLP-Supplementary-Materials, and the experiment was not preregistered.
